# The effect of lesser mealworm protein on exercise-induced muscle damage in active older adults: a randomized controlled trial

**DOI:** 10.1016/j.jnha.2024.100204

**Published:** 2024-03-08

**Authors:** Lotte Koopmans, Marcia Spoelder, Coen C.W.G. Bongers, Thijs M.H. Eijsvogels, Maria T.E. Hopman

**Affiliations:** a Department of Medical BioSciences, Radboud University Medical Center, Nijmegen, The Netherlands; b Department of Primary and Community Care, Radboud University Medical Center, Radboud, The Netherlands; c School of Sport and Exercise, HAN University of Applied Sciences, Nijmegen, The Netherlands

**Keywords:** Elderly, Muscle damage, Endurance exercise, Insect, Alphitobius diaperinus

## Abstract

**Objectives:**

We compared the effect of 12 weeks lesser mealworm-based (*Alphitobius diaperinus*) protein supplementation to whey protein and placebo supplementation on Exercise-Induced Muscle Damage (EIMD) after long-distance walking in older adults.

**Methods:**

in this randomized controlled trial, 70 physically active older adults (>60 years) were randomly allocated to the following groups: I) lesser mealworm protein, II) whey protein or III) iso-caloric placebo. Participants received supplements 11 weeks before and 1 week during a 3-day long-distance walking challenge (30−50 km per day). Blood concentrations of creatinine kinase (CK) and lactate dehydrogenase (LDH), handgrip strength and muscle soreness were measured pre-exercise and directly after each walking bout.

**Results:**

Significant elevations of CK concentrations (103 [76–161] U/l to 758 [342–1104] U/l, p < 0.001) and LDH concentrations (202 [175–220] to 283 [252–339] U/l, p < 0.001) were observed following 7h45 min ± 11 min of walking exercise per day, but the magnitude of this effect did not differ among suppletion groups. Hand grip strength decreased significantly (p < 0.001) while muscle soreness increased (p = 0.002) after the first walking day compared to pre-exercise, with no group differences.

**Conclusion:**

12-weeks of lesser mealworm-based protein supplementation (30 g/day) does not attenuate exercise induced muscle damage in older adults following three days of prolonged walking exercise in comparison to placebo or whey protein.

## 1 Introduction

Physical activity may lead to micro injuries of contractile proteins in the skeletal muscle of older adults, also known as exercise-induced muscle damage (EIMD) [1]. EIMD leads to transient loss of muscle function and delayed onset of muscle soreness [2], which may hamper older adults to maintain an active lifestyle. Previous studies showed positive effects of protein ingestion on reducing EIMD following both long and short duration exercise bouts in older and younger adults [[3], [4], [5]]. A protein intake between 1.2 and 2.0 g/kg/day is recommended for physically active older adults [6], but more than 50% of these older adults do not reach those threshold values [7].

The world’s increasing population challenges the availability of sufficient, high-quality dietary protein resources in the future. Insects may be a promising protein source since its well-balanced amino acid composition meets the essential amino acid requirements needed for muscle protein synthesis [6,8], similar to the amino acid composition of whey protein [9]. While previous studies have shown the potential of whey protein to attenuate EIMD [5], the effectiveness of insect-based protein supplements, such as lesser mealworm, on EIMD has not been examined yet.

This randomized double-blind placebo-controlled trial aimed to assess the effect of lesser mealworm (*Alphitobius diaperinus)* versus whey protein supplementation in comparison to placebo on EIMD markers in physically active older adults during 3 consecutive days of long-distance walking exercise. Secondary outcomes included differences among groups in exercise-induced muscle soreness and changes in handgrip strength. We hypothesized that lesser mealworm and whey protein supplementation will attenuate EIMD compared to the placebo group.

## 2 Methods

### 2.1 Participants

Participants were recruited in March 2022 via the Nijmegen Exercise Study database [10]. Individuals aged ≥60 years, who registered for the Nijmegen Marches 2022 and were able to walk ≥30 km for multiple consecutive days were eligible for inclusion. Exclusion criteria were allergic or sensitive for milk proteins, shell or shellfish, lactose intolerance, a BMI > 30 kg/m^2^, Chronic Obstructive Pulmonary Disease (COPD), renal insufficiency, cancer treatment, use of statins, or intestinal diseases that may influence protein uptake. Moreover, consumption of other protein supplements or performance of resistance exercise training was not allowed during the study period. All participants provided written informed consent prior to any experimental procedure. The study conformed to the principles of the Declaration of Helsinki, was approved by the local Medical Ethical committee (Study-ID: NL79716.091.21) and registered at the Dutch trial registry (NL9862).

### 2.2 Study design

**Fig. 1:**
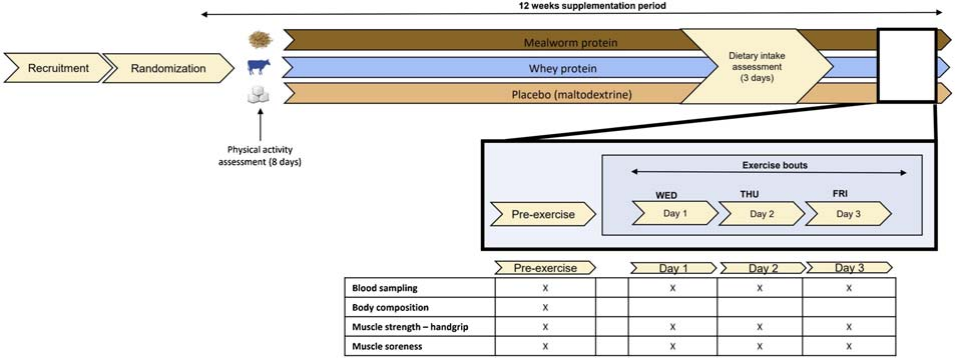
Study Design. Overview of study timeline and measurements included in this study.

In this randomized double-blind placebo-controlled trial a total of 70 participants were recruited and randomly allocated to a lesser mealworm protein-, whey protein or placebo supplement group. Participants were invited for five study visits (Fig. 1). Measurements were performed upon randomization, at pre-exercise (after 11 weeks protein supplementation) and within 30 min after completion of each exercise bout (for 3 consecutive days) during the 12th week of supplementation.

### 2.3 Protein intervention

Participants were instructed to consume the assigned supplement every day for a period of 12 subsequent weeks. The supplements were provided as a dried powder in blinded and labelled sachets with unique charge numbers. The supplement powder had a chocolate-coconut taste and had to be dissolved in a liquid (e.g. water, juice) and ingested by the participant. Participants were instructed to consume one sachet in the morning and another sachet in the afternoon/evening, resulting in a total of 31 g, 32 g and 3 g additional protein intake per day for respectively the lesser mealworm, whey and placebo group (Table 1). The small amount of protein in the placebo sachet was due to the coconut flakes in the shake to ensure blinding of the supplements. All supplements were supplied by YNSECT Nutrition & Health (Ermelo, The Netherlands). Protein supplements were produced according to the HACCP/ISO22000 regulations at certified companies and were made from approved and commercially available ingredients. Participants were asked to report their supplement intake every day by filling in a diary.

**Table 1:** Nutritional composition of whey, lesser mealworm and placebo supplements.

Nutrient	Lesser mealworm Protein	Whey Protein	Maltodextrin (Placebo)
Energy (kcal/100 g)	467	463	491
Fat (g/100 g)	26.3	23.5	27.0
Carbohydrate (g/100 g)	6	14.2	54.0
Of which sugar (g/100 g)	2.2	4.3	4.9
Protein (g/100)	48.0	47.5	4.2
			
Amino Acid content (mg/100 g)			
Alanine	3100	2800	10
Arginine	2500	1300	15
Aspartanic Acid	3800	5800	23
Cystine	400	1300	4
Glutamic Acid	5700	9200	38
Glycine	2100	900	10
Histidine	1400	900	3
Isoleucine^a^	1900	3600	9
Leucine^a^	3000	3600	14
Lysine	3000	5100	8
Methionine	600	1100	3
Phenylalanine	1900	1600	11
Proline	3000	3300	11
Serine	1900	3000	12
Threonine	1800	3900	10
Tryptophane	500	800	3
Tyrosine	3000	200	3
Valine^a^	2500	3400	14

a Branches Chain Amino Acids (BCAA’s).

## 3 Measurements

### 3.1 Habitual physical activity and exercise training

Habitual physical activity was measured upon randomization using an ActivPAL monitor (PAL technologies Ltd, Glasgow, UK) [11] for 8 consecutive days within the first month of supplementation. The small device (25 × 45 × 5 mm) was attached to the upper thigh. A self-reported sleep diary in combination with a validated algorithm was used to identify sitting, standing and stepping during wear-time [12]. A measurement day was considered invalid when (1) a single activity took up more than 95% of total awake time, (2) step count was below 1000 or 3) the number of awake hours was less than 10. A valid habitual physical activity assessment consisted of ≥5 valid days, including at least 1 weekend day. Outcomes were reported as sedentary time (h/day), step count (n/day), time spent in light intensity physical activity (LIPA, min/day) and time spent in moderate-to-vigorous physical activity (MVPA, min/day) [13]. Participants also kept track of their walking training sessions during the 11 weeks of supplementation and accordingly cumulative walking kilometers during the supplementation period were calculated.

### 3.2 Anthropometrics and muscle mass

Height, weight, body composition and waist- and hip circumference of the participants were measured pre-exercise. Waist-hip ratio was subsequently calculated. Total skeletal muscle mass (SMM) was assessed with bioelectrical impedance analyses (InBody 770 Body Composition Analyzed, Seoul, South Korea) [14]. Participants were instructed not to eat or drink for 2 h prior to the measurement and were asked to empty their bladder shortly before their body composition measurement.

### 3.3 Dietary intake

Food consumption patterns were assessed the week before the pre-exercise visit by using an online Dutch tool (i.e., ‘*Mijn Eetmeter’)* [15]. Participants entered their full eating and drinking pattern for 3 days, with at least one weekend day. The nutritional composition of the supplement was manually added to each dietary intake file by the researcher after unblinding. The average total energy intake, as well as the protein intake were subsequently calculated.

### 3.4 Exercise intervention

Participants participated in a mass-participation walking-exercise event (Nijmegen Marches, https://www.4daagse.nl/en) with daily organized walking bouts of 30, 40 or 50 km at a self-selected pace. Walking distance and exercise duration were registered directly following each walking bout, and walking speed was calculated accordingly.

### 3.5 Blood samples

Venous blood was drawn from the antecubital vein during each visit, and serum and lithium heparin plasma samples were stored at −80 °C until further analyses. Creatine kinase (CK) and lactate dehydrogenase (LDH) concentrations were measured by trained technicians using standard operating procedures on an Atellica CH Analyzer, (Siemens, Erlangen, Germany) to identify muscle damage and tissue damage, respectively.

### 3.6 Handgrip strength

Handgrip strength of the right hand was measured with a hydraulic, analogue handheld dynamometer (JAMAR®, Chicago, IL, USA). For every participant, the dynamometer was adjusted to their hand size. The participants were seated in a chair with the elbow flexed in a 90-degree angle position. Arm support by chair was not allowed. Participants were asked to shortly squeeze the handgrip instrument as hard as they could for three times, with one minute rest in between each measurement [16]. Maximum strength in kilograms was used for analysis.

### 3.7 Muscle soreness

Muscle soreness was assessed using a Numeric Pain Rating Scale (NPRS) where participants could mark a pain score (0 = no muscle soreness, 10 = extreme muscle soreness). NPRS scores of 1–5 were considered as mild muscle soreness, 6-7 as moderate muscle soreness and >7 as severe muscle soreness [17,18].

## 4 Statistical analysis

Statistical analyses were performed using SPSS software (IBM SPSS Statistics for Windows, Version 25.0 IBM Corp., Armonk, NY, USA) and graphs were made using Graphpad Prism 9 (Boston, USA). All continuous variables and the residuals of the variables used in the linear-effects model were visually inspected and tested for normality with the Kolmogorov-Smirnov test. Logarithmic transformations were applied when data were not normally distributed and subsequently the normality was retested. The data of logarithmic transformations of CK, LDH and handgrip strength were used in the statistical analyses. Data were displayed as mean ± SD or median (interquartile range [IQR]) for parametric and non-parametric continuous variables, respectively. Group differences were analyzed using a One-Way ANOVA or Kruskal Wallis for parametric or non-parametric continuous variables, respectively. Repeated observations over days, such as CK, LDH and handgrip strength, were analyzed using linear mixed models with pre-exercise values as covariate. When significant main effects or interactions were detected, Bonferroni post-hoc comparisons were made in case of parametric variables and Mann-Whitney U tests in case of non-parametric variables. The level of significance was set at p < 0.05 (two-sided).

## 5 Results

### 5.1 Participants

A total of 70 older adults were recruited of which 59 participants completed the 11-week protein upload protocol and started the mass-participation walking-exercise event. Drop-out during the supplementation period was significantly higher in the lesser mealworm group compared to the whey protein and placebo group (p = 0.026). Six participants refrained from further consumption due to physical symptoms such as diarrhea, loss of appetite, nausea and floated stomach feeling (lesser mealworm n = 4; placebo n = 2), whereas three participants stopped the consumption due to dislike of the supplement taste (lesser mealworm n = 2; whey n = 1) and two participants withdrew due to personal reasons (lesser mealworm n = 1; whey n = 1). During the mass-participation walking-exercise event, 5 participants dropped out following the first (lesser mealworm n = 3) or second walking day (lesser mealworm n = 1; whey n = 1) due to an injury or personal reason, leaving 54 participants who completed the entire study (lesser mealworm n = 12; whey n = 21 and placebo n = 20) (Supplemental Fig. S1). Participants (51% male), who finished the 11-week protein upload protocol were 69 ± 5 years old with a BMI of 24.6 ± 3.1 kg/m^2^. No differences were observed for demographics and anthropometrics among the three groups (Table 2).

**Table 2:** Pre-exercise characteristics of the total group and specified for the lesser mealworm-, whey- and placebo supplement group.

	Total group n = 59	Lesser mealworm n = 16	Whey n = 23	Placebo n = 20	p-value
Demographics					
Age (years)	69 ± 5	68 ± 4	70 ± 5	68 ± 5	0.25
Male, n (%)	30 (51)	9 (56)	12 (52)	9 (45)	0.79
Anthropometrics					
Body weight (kg)	72.2 ± 12.5	75.2 ± 13.2	72.5 ± 12.0	69.9 ± 12.7	0.48
Height (m)	1.72 ± 0.10	1.72 ± 0.10	1.72 ± 0.10	1.70 ± 0.10	0.82
BMI (kg/m^2^)	24.5 ± 2.6	25.6 ± 2.8	24.2 ± 2.1	23.9 ± 2.7	0.12
Waist-hip ratio	0.91 ± 0.10	0.94 ± 0.10	0.92 ± 0.10	0.89 ± 0.10	0.24
Skeletal muscle mass (kg)	29.5 ± 6.1	30.4 ± 6.2	29.7 ± 6.1	28.6 ± 6.2	0.61
Dietary intake					
Energy intake (kcal)	1905 ± 420	1868 ± 359	2029 ± 403	1791 ± 464	0.18
Protein intake <1.2 g/kg/day (%)	25 (45)	7 (50)	3 (13)	15 (75)	**<0.001**
Protein intake (g/kg/d)	1.3 ± 0.4	1.4 ± 0.4	1.5 ± 0.3	1.0 ± 0.3	**<0.001**
Physical activity					
Sedentary time (h/day)	9.0 ± 1.8	9.4 ± 2.2	9.1 ± 1.3	8.4 ± 2.0	0.35
Step count (N/day)	7336 [5993−9369]	6599 [5678−9537]	7444 [6005−9004]	7502 [5744−10263]	0.82
MVPA (min/day)	115 [94−145]	108 [86−145]	115 [96−147]	121 [85−159]	0.85
LIPA (min/day)	259 [193−335]	267 [182−350]	257 [192−304]	250 [196−367]	0.89
Cumulative walking distance during supplementation period	460 [323–671]	462 [346−742]	594 [395−729]	450 [335−761]	0.47
Exercise distance bout per day					
30 km (%)	43 (73)	11 (69)	17 (74)	15 (75)	
40 km (%)	12 (20)	4 (25)	4 (17)	4 (20)	0.97
50 km (%)	4 (7)	1 (6)	2 (9)	1 (5)	

Data are presented as number (with percentage between brackets) of participants, mean ± SD for parametric data or median [interquartile range] for non-parametric data. Body Mass Index (BMI), h = hours. n = number; min = minutes; MVPA = moderate to vigorous physical activity; LIPA = light intensity physical activity.

#### 5.1.1 Habitual dietary intake

Protein intake during the suppletion period was higher in the lesser mealworm (1.4 ± 0.4 g/kg/day) and whey protein group (1.5 ± 0.3 g/kg/day) compared to the placebo group (1.0 ± 0.3 g/kg/day, p < 0.001). The average energy intake was 1905 ± 420 kcal/day and did not differ among groups (p = 0.18).

#### 5.1.2 Physical activity

Four ActivPAL measurements were missing due to devices’ error, leaving data of 55 participants. Participants spent 115 [94−145] min/day MVPA and 259 [193−335] min/day LIPA, step count was 7,336 [5,993−9,369] and sedentary time was 9.0 ± 1.8 h per day. No differences among groups were observed for MVPA, LIPA, step count and sedentary time (p = 0.85, p = 0.89 and p = 0.82, p = 0.35, respectively, Table 2). The cumulative walking distance during the supplementation period was 460 [323–671] kilometers, with no group differences (p = 0.53).

### 5.2 Exercise characteristics

The majority of participants walked 30 km per day (n = 43; 73%, Table 2). Average walking speed was lower at the first walking day (3.9 ± 3.1 km/h) compared to the second (4.5 ± 0.73 km/h) and third day (4.5 ± 0.8 km/h) (P_time_<0.001). There were no group differences or interaction effects in walking speed for the three walking days (P_group_ = 0.62, P_time*group_ = 0.64). On average, participants walked 7h45 min ± 11 min per day.

### 5.3 Muscle damage

**Fig. 2:**
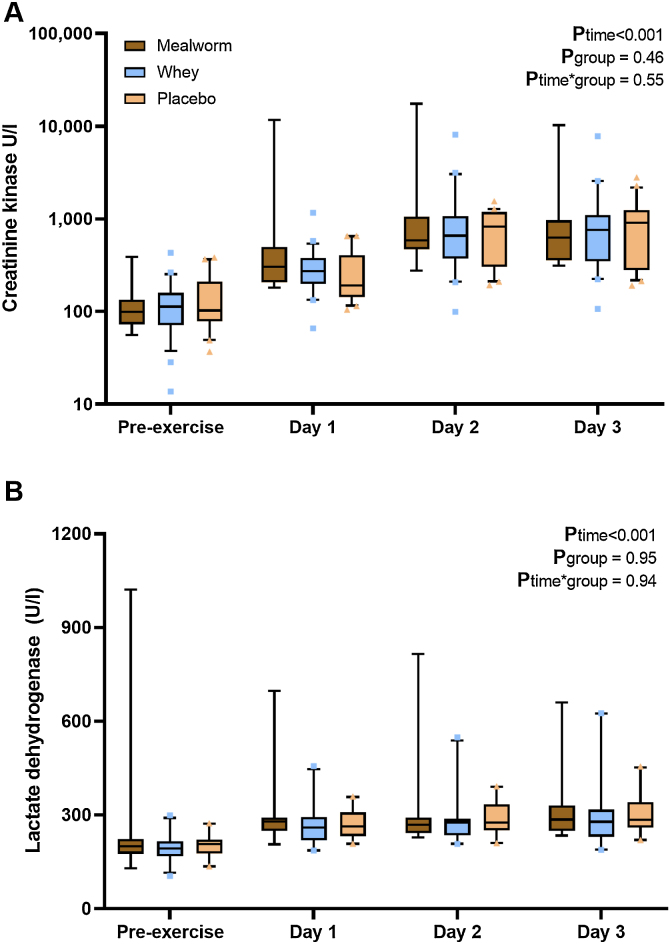
Muscle damage markers. Box-and-whisker plots for creatinine kinase (CK) (A) and lactate dehydrogenase (LDH) (B). The box-and-whisker plots represent the median, interquartile range, 5–95% percentile (upper and lower whiskers) and outliers (dots). Prolonged walking resulted in elevated CK and LDH concentrations in all groups (p < 0.001). No significant differences in exercise-induced responses were observed among suppletion groups (all p > 0.05).

Creatinine kinase (CK) concentrations significantly increased from 103 [76–161] U/l pre-exercise to 217 [163–374] U/l, 662 [379–1130] U/l and 758 [342–1104] U/l on day 1, day 2 and day 3, respectively (P_time_ <0.001) (Fig. 2A). The magnitude of exercise-induced increases in CK concentrations did not differ among suppletion groups (P_time*group_ = 0.55).

Lactate dehydrogenase (LDH) concentrations significantly increased from 202 [175–220] pre-exercise to 264 [235–296] U/l, 275 [243–300] U/l and 283 [252–339] U/l on day 1, day 2 and day 3, respectively (P_time_ <0.001) (Fig. 2B**)**. The magnitude of exercise-induced increases in LDH concentrations did not differ among suppletion groups (P_time*group_ = 0.94).

### 5.4 Handgrip strength

**Fig. 3:**
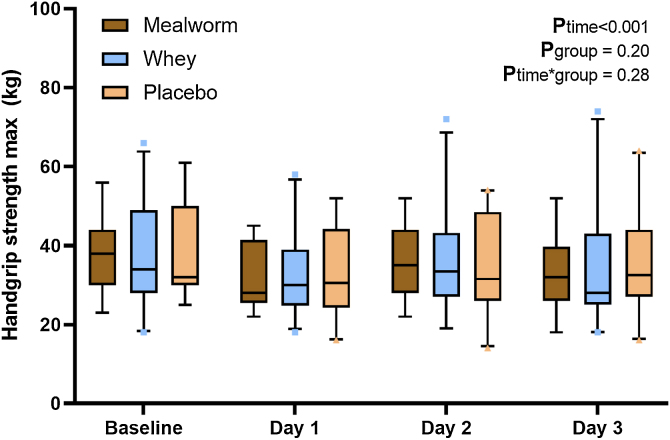
Handgrip strength. Box-and-whisker plots for handgrip strength. The box-and-whisker plots represent the median, interquartile range, 5–95% percentile (upper and lower whiskers) and outliers (dots). No significant differences were observed among the groups at the different time points (all p & 0.05).

Handgrip strength significantly decreased from pre-exercise to post-exercise at day 1 (p < 0.001), whereas no differences in handgrip strength were observed between pre- and post-exercise assessments at day 2 and 3 (Fig. 3). Changes in handgrip strength were not different among groups (P_group*time_ = 0.28).

### 5.5 Muscle soreness

**Fig. 4:**
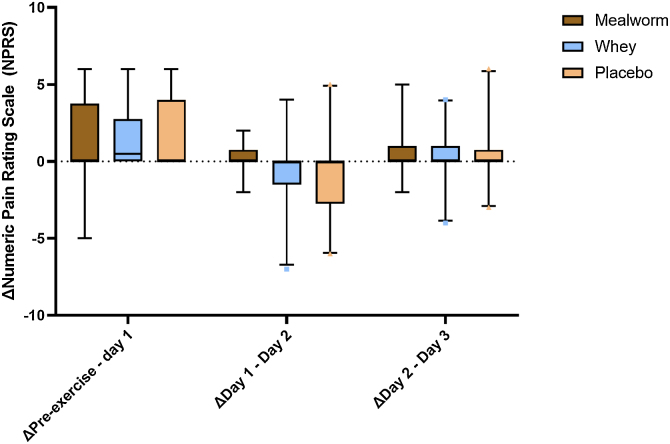
Changes in muscle soreness. Box-and-whisker plots for changes in muscle soreness. The box-and-whisker plots represent the median, interquartile range, 5-95% percentile (upper and lower whiskers) and outliers (dots). No group differences were observed in changes in muscle soreness during this study.

Muscle soreness increased from pre- to post-exercise at day 1 (p = 0.002) and remained elevated during post-exercise assessments at day 2 and day 3. Daily changes in muscle soreness did not differ among groups (P_Δpre-exercise – day 1_ = 0.79; P_Δday1-day2_ = 0.42; P_Δday2-day3_ = 0.98) (Fig. 4).

## 6 Discussion

To our knowledge, this is the first study to assess the effects of 12 weeks of lesser mealworm protein supplementation on EIMD in physically active older adults. Walking exercise induced a significant rise in CK and LDH levels, however, the magnitude of exercise-induced CK and LDH responses was comparable among the lesser mealworm protein, whey protein and placebo group. Moreover, we found no differences in changes of muscle soreness and handgrip strength among suppletion groups. These findings are contradictory to our hypothesis and suggest that the adopted protein supplementation protocols were insufficient to reduce muscle damage markers after repeated bouts of long-distance walking exercise in physically active older adults.

In contrast to our hypothesis, lesser mealworm nor protein supplementation did not attenuate EIMD. A potential explanation for this neutral finding may be a limited biologic availability of amino acids. However, two previous studies examined time-dependent changes in amino acid concentrations after the intake of lesser mealworm proteins and found no difference in the biological availability of amino acids based on the magnitude of increases in amino acid concentrations compared to intake of soy protein or milk proteins [19,20]. Furthermore, the amino-acid composition of our lesser mealworm and whey protein supplement was comparable (Table 1), and several previous studies showed that supplementation of such high quality proteins could attenuate EIMD in younger and older adults [5,[21], [22], [23], [24]].

A previous study which used a similar experimental protocol as the current study with repetitive prolonged-walking exercise bouts and a 12-week upload period showed no effect of whey protein supplementation on EIMD either [25]. The lack of a positive effect of protein supplementation on EIMD might be explained by the fact that muscle damage markers reach peak concentrations 24 h–72 h post-exercise [5,25,26], whereas muscle protein synthesis occurs directly after muscle damage [1]. This means that on the second and third day of walking, both the release of muscle damage markers and protein synthesis will have occurred. Thus, data in our study represent the combined effect of both processes, and although reflecting a real-life situation, where physical exercise is a daily habit of older adults, the effect of protein supplementation maybe masked.

Another potential explanation for our neutral findings is that most positive studies on the effects of protein supplementation on EIMD were conducted in younger adults [[21], [22], [23], [24]]. It is well known that older adults are less responsive to the anabolic stimulus of protein than younger individuals [27]. Sufficient protein intake is essential for muscle protein synthesis and improvement of muscle mass and strength [28]. A protein intake of 1.0–1.2 g per kilogram body weight per day (g/kg/day) is recommended for elderly [28]. Despite the supplementation of >30 g of protein/day and reaching average levels of protein intake >1.2 g/kg/day in both lesser mealworm and whey protein groups, individual data showed that 50% of the participants within the lesser mealworm and 14% in the whey protein supplementation group did not reach levels above 1.2 g/kg/day. These results are comparable to other protein supplementation studies in older adults where the mean protein intake remained below 1.2 g/kg/day [26,29]. Future studies should, therefore, consider a higher protein supplementation dosage because of the anabolic resistance in elderly.

Muscle soreness increased and handgrip strength decreased from pre-exercise to the first day of walking, but these changes did not differ among groups. It is well known that EIMD leads to loss of muscle strength and increased muscle soreness in the first 48 h after exercise [1]. Previous studies showed similar effects of protein supplementation compared to placebo on muscle soreness and muscle strength after exercise [3,5,30]. One may suggest that the hand grip strength protocol that we adopted may not be sensitive enough to detect changes in lower limb strength, but previous studies performing lower limb muscles measurements could not find differences either [3,30]. Therefore, it seems that protein supplementation does not impact muscle soreness and handgrip strength compared to placebo after a three days walking bout.

Our study has some limitations. First, walking exercise induced a significant increase in CK concentrations, but the magnitude of this increase might not be large enough to show differences across the supplements that we investigated. Our study showed median CK concentrations up to 758 [342–1104] U/l whereas previous step and resistance exercise studies showed CK concentrations up to 7239 ± 2403 U/l [[31], [32], [33]]. Second, the extrapolation of our findings to the general population may be limited since this study had an exercise protocol consisting of long-distance walking bouts (≥30 km) for 3 consecutive days. Future studies should, therefore, also investigate the benefits of protein supplementation on more real-

life physical activities in older adults. Finally, muscle strength was assessed using hand grip strength measurements. This method is well accepted to quantify muscle strength and general condition [[34], [35], [36]], but future studies may consider additional leg strength testing as a more sensitive approach to assess changes in functional muscle characteristics following prolonged walking exercise.

In conclusion, 12-weeks of lesser mealworm-based protein supplementation (30 g/day) does not attenuate exercise induced muscle damage in older adults following three days of prolonged walking exercise in comparison to placebo or whey protein.

## Conflicts of interest

The results of the study are presented clearly, honestly, and without fabrication, falsification, or inappropriate data manipulation. The authors declare no conflict of interest.
