# Supplementary material for: The effect of lesser mealworm protein on exercise-induced muscle damage in active older adults: a randomized controlled trial
Source: J Nutr Health Aging. 2024 Mar 8;28(5):100204. doi: 10.1016/j.jnha.2024.100204 (PMC12877278; doi:10.1016/j.jnha.2024.100204)
Supplement: Supplementary file 1 [file mmc1.docx]

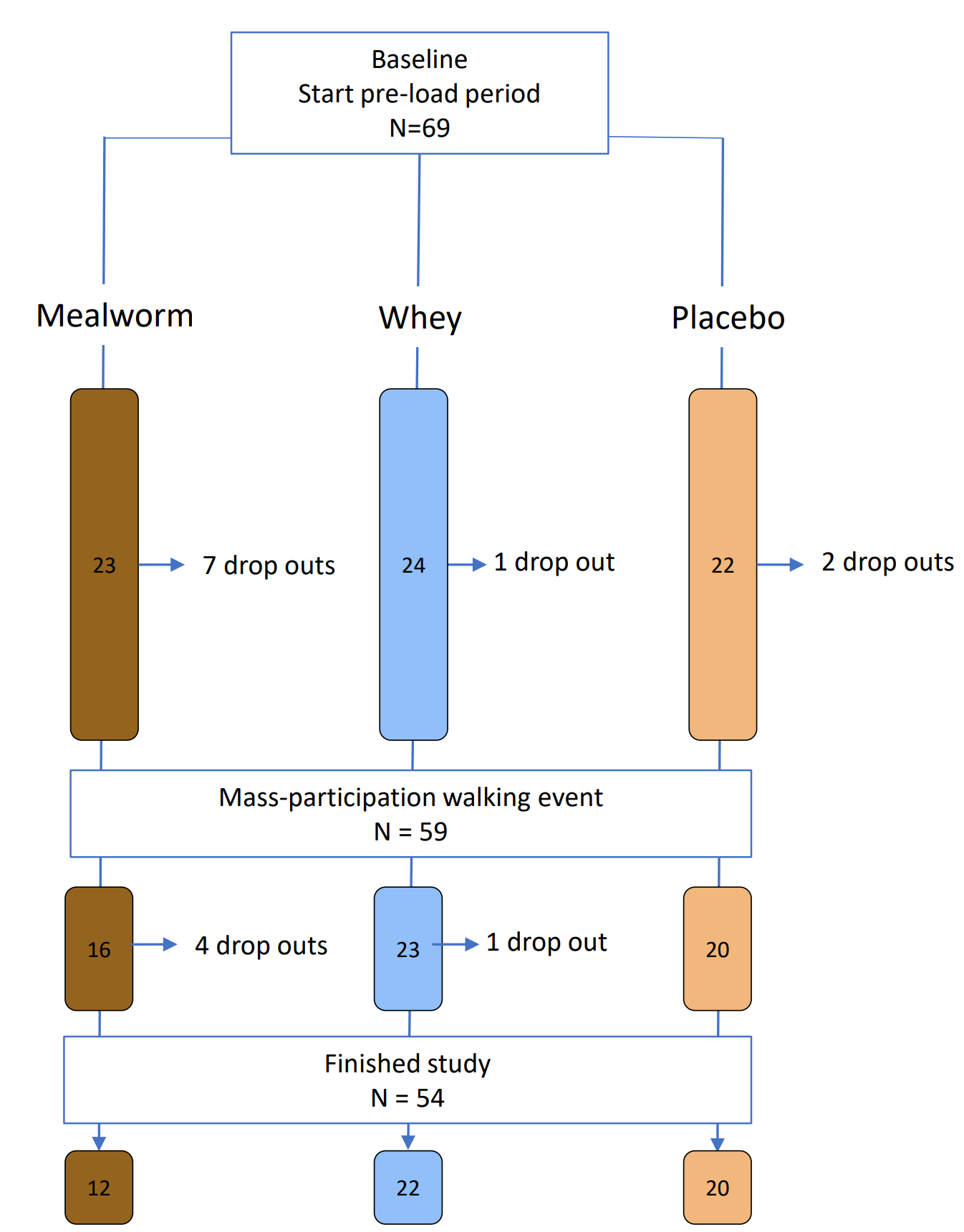


**Supplemental figure 1| Flow Chart drop-out.** Overview of the drop-outs during the study. The lesser mealworm group had a significantly higher drop-out (p = 0.027) compared to the whey protein and placebo group. Drop-outs were mostly because of health symptoms and dislike of taste.
